# Association between heavy metal and metalloid levels in topsoil and cancer mortality in Spain

**DOI:** 10.1007/s11356-017-8418-6

**Published:** 2017-01-20

**Authors:** Olivier Núñez, Pablo Fernández-Navarro, Iván Martín-Méndez, Alejandro Bel-Lan, Juan F. Locutura Rupérez, Gonzalo López-Abente

**Affiliations:** 1 Environmental and Cancer Epidemiology Unit, National Centre for Epidemiology, Carlos III Institute of Health, Avda. Monforte de Lemos 5, 28029 Madrid, Spain; 2 Consortium for Biomedical Research in Epidemiology and Public Health (CIBER en Epidemiología y Salud Pública - CIBERESP), Madrid, Spain; 3 Department of Geochemistry and Mineral Resources, Spanish Geological and Mining Institute (Instituto Geológico y Minero de España/IGME), Ríos Rosas, 23, 28003 Madrid, Spain

**Keywords:** Cancer mortality, Spatial data analysis, Geochemistry, Soil composition, Compositional analysis, Medical geology

## Abstract

Spatio-temporal cancer mortality studies in Spain have revealed patterns for some tumours which display a distribution that is similar across the sexes and persists over time. Such characteristics would be common to tumours that shared risk factors, including the geochemical composition of the soil. The aim of this study was to assess the possible association between heavy metal and metalloid levels in topsoil (upper soil horizon) and cancer mortality in mainland Spain. Ecological cancer mortality study at a municipal level, covering 861,440 cancer deaths (27 different tumour locations) in 7917 Spanish mainland towns, from 1999 to 2008. The elements included in this analysis were Al, As, Cd, Cr, Cu, Fe, Mn, Ni, Pb and Zn. Topsoil levels (partial extraction) were determined by ICP-MS at 13,317 sampling points. For the analysis, the data on the topsoil composition have been transformed by the centred logratio (clr-transformation). Principal factor analysis was performed to obtain independent latent factors for the transformed variables. To estimate the effect of heavy metal levels in topsoil composition on mortality, we fitted Besag, York and Mollié models, which included each town’s factor scores as the explanatory variable. Integrated Nested Laplace Approximation (INLA) was used as a tool for Bayesian inference. All results were adjusted for sociodemographic variables. The results showed an association between trace contents of heavy metals and metalloids in topsoil and mortality due to tumours of the digestive system in mainland Spain. This association was observed in both sexes, something that would support the hypothesis that the incorporation of heavy metals into the trophic chain might be playing a role in the aetiology of some types of cancer. Topsoil composition and the presence of potentially toxic elements in trace concentrations might be an additional component in the aetiology of some types of cancer, and go some way to determine the ensuing geographic differences in mortality in Spain. The results support the interest of inclusion of heavy metal levels in topsoil as a hypothesis in analytical epidemiological studies using biological markers of exposure to heavy metals and metalloids.

**Electronic supplementary material:**

The online version of this article (doi:10.1007/s11356-017-8418-6) contains supplementary material, which is available to authorized users.

## Introduction

The study of the relationship between natural geological factors and health in humans and animals has been called medical geology (Selinus et al. 2013). It is a field of research with many years of tradition, which forms part of the discipline known as environmental epidemiology. Historically, it arose as a result of the observation of geographical differences in disease distribution (Peeters 1987). Medical geology is currently undergoing a qualitative change due to the interaction among scientist from different disciplines, and in research opportunities to better understand how the natural environment impacts human health (Centeno et al. 2013).

The Spanish Geological and Mining Institute (*Instituto Geológico and Minero de España/IGME*) recently published the “Geochemical Atlas of Spain”, the first geochemical study of surface materials to cover the entire country (Locutura et al. 2012), and the study reported here is fruit of the collaboration among cancer epidemiologists (López-Abente et al. 2007) (López-Abente et al. 2014) and geochemists from IGME (Locutura et al. 2012).

The presence of toxic metals in soil per se, and in soil impacted human activities is a major concern for both human health and ecotoxicology (Ranville 2005). Exposure to arsenic and heavy metals is associated with various types of cancer, but there is far less information on the health effects of low-dose chronic exposure to many trace metals (Naujokas et al. 2013) (Centeno et al. 2013). Studies on the health effects of metals in topsoil belong to this latter category.

Data drawn from geochemical soil studies tend to be recorded in milligram per kilogram or parts per million (ppm) and have been called compositional data (Aitchison 1994) or closed number systems (Reimann et al. 2011). Data of this type are characterised by vectors whose components are the part of a whole and are not therefore independent (e.g. their sum is a constant). For instance, in geochemical soil studies, this could induce negative correlations or no correlations in variables one would logically expect to be positively correlated. The usual approach in the compositional analysis is to remove the closure effects in data points, using different transformations, (viz, logratio analysis) (Aitchison 1982) based on relative magnitudes. Because the absolute magnitudes of compositional variables are ratios to a common sum, Aitchison proposed to use relative magnitudes by calculating the ratio of each compositional variable compared to a single variable that functions as a constant divisor (Xie et al. 2003). Multivariate analysis is applied to logratios instead of the original data.

In this context, the aim of this study was to assess the possible association between heavy metal and metalloid levels in topsoil and mortality due to 27 different tumour types in 7917 Spanish mainland towns, with the resulting spatial relative risk (RR) estimates being adjusted for sociodemographic variables as possible confounders.

## Material and methods

### Mortality data and soil sampling and metal analysis

A detailed description of the data of mortality and taken soil samples can be found in a previously published study (Nuñez et al. 2016). Briefly, mortality data (observed and expected cases) for each of the 8077 (7917 mainland) Spanish towns were drawn from the records of the National Statistics Institute (NSI) for the study period (1999–2008) and computed for 27 types of malignant tumours (see Supplementary data, Table S1) with a total of 861,440 deaths due to the tumours analysed. Across the period June 2008–November 2010, a total of 21,187 residual soil samples (13,505 from the surface horizon and 7682 from the deeper horizon) were collected at a total of 13,505 sampling points (13,317 in mainland Spain and 188 on the Canary and Balearic islands). Residual soil is a soil belonging to the geological substratum and therefore not transported. The residual soil samples were analysed by instrumental inductively coupled plasma mass spectrometry (ICP-MS). The elements included in this analysis were Al, As, Cd, Cr, Cu, Fe, Mn, Ni, Pb and Zn. A detailed description of the sample-collection and the chemical-analysis techniques used can be found in the Geochemical Atlas of Spain (Locutura et al. 2012). All the laboratory determinations were performed at Activation Laboratories Ltd., (Actlabs, Ontario).

Cancer mortality data are aggregated at a town area level, while the data concentrations of metals in the soil are measures taken at sampling locations across the country. In order to obtain a representative value of this concentration at the town area level, an interpolation method (ordinary kriging) for the town’s centroid was used (Ribeiro and Diggle 2001) (Diggle and Ribeiro 2006).

### Topsoil data transformations

For compositional data, the sum of all concentrations of the elements in each sample is almost constant or at least restricted. Then to avoid spurious correlation, the soil composition estimated at each town were transformed by centred logratio transformation (clr-transformation). The clr-transformation (Filzmoser et al. 2010) (Aitchison 1982) (Aitchison 2003) results in a multivariate observation *y* = (*y*_1_, ... *y*_D_), and is defined as:

$$ y=\left(\mathit{\log}\frac{x_1}{\sqrt{\prod_{i=1}^D{x}_i}}\dots, \mathit{\log}\frac{x_D}{\sqrt{\prod_{i=1}^D{x}_i}}\right) $$

Each value of a variable for each point was divided by the geometric mean of all variables for that point, and the logarithms then obtained (Reimann et al. 2011).

### Reduction of dimensions (factorial analysis)

A direct consequence of the previous transformation is emerging collinearity (e.g. cadmium occurs mainly in ores with zinc and to lesser degree with lead and copper). Moreover, clr-transformation produces variables whose correlation matrix is singular. It is therefore difficult to conduct a regression analysis with such explanatory variables.

In order to avoid this problem, a factorial analysis was conducted to obtain independent latent factors for the clr-transformed variables (Filzmoser et al. 2010). This type of analysis provides information about the internal structure of the geochemical data, reduces data dimensionality to a few representative factors and thus seeks to summarise the multivariate information in a compact form. We performed this analysis by using robust principal factor analysis (PFA), in which 4 factors obtain a cumulative variance of over 75%. For statistical analysis purposes, the factor scores for each point were extracted after rotation by the varimax method.

### Statistical analysis

The association between metal concentrations in soil and cancer mortality was assessed in an ecological regression, where the response was the number of observed deaths from cancer, with expected cases as offset, and the exposure covariate were the factor scores obtained from the kriging estimate of the metal concentration in the municipal-centroid area.

Let *F*_*ij*_ denote the factorial burden for each factor (*j*) at each centroid area location (*i*). Assume that the observed number of cases *O*_*i*_ in the *I* ^th^ area is Poisson distributed, with mean *E*_*i*_ *λ*_*i*_, where *E*_*i*_ is the expected number of cases in that area and the relative risk *λ*_*i*_ follows a log-linear model, such that:

$$ \mathit{\log}\left({\lambda}_i\right)=\alpha +\sum {{}_j^4}_{=1}{\beta}_j{F}_{i j}+\sum_k{\delta}_k{Soc}_{i k}+{u}_i+{v}_i $$

where *α *is an intercept, *β*_*j*_ is the coefficient for the exposure covariates *F*_*ij*_ obtained from the factorial analysis, *Soc*_*ik*_ sociodemographic indicators, *v*_*i*_ are unstructured normal residuals and *u*_*i*_ are spatially structured effects which follow an intrinsic conditional autoregressive model, namely, the Besag, York and Mollié model (BYM) (Besag et al. 1991). Inference for the parameters of interest is made in a Bayesian framework, and prior distributions are specified for all parameters.

The sociodemographic indicators (Soc_*ik*_ *)* were obtained from the 1991 census and considered for their availability at the city level and potential explanatory ability vis-à-vis certain geographic mortality patterns (López-Abente et al. 2006). These indicators were as follows: population size (categorised into three levels 0–2000 [rural zone], 2000–10,000 [semi-urban zone], and over 10,000 inhabitants [urban zone]); percentages of illiteracy, farmers and unemployment; average number of persons per household and mean income.

All the analysis and graphic representations has been effected with the program R and their different libraries in each case, the kriging with geoR (Ribeiro and Diggle 2001) and factorial analysis with StatDA (Reimann et al. 2011). Integrated Nested Laplace Approximations (INLAs) (Rue et al. 2009) were used as a tool for Bayesian inference. For this purpose, we used R-INLA (Rue and Martino 2010), with the option of “Gaussian” estimation of the parameters. A total of 7917 Spanish mainland towns were included, and the spatial data on municipal contiguities were obtained by processing the official NSI maps.

## Results

The mean topsoil concentrations in towns in the study area are shown in Table 1. Soil levels ranged from 0.01 to >5000 mg kg^−1^ for cadmium and lead, and from 0.10 to >2000 mg kg^−1^ for arsenic, nickel and lead. The interpolation procedure reduced the range of determinations in both elements, basically influencing the extreme values.

**Table 1:** Study of topsoil metal levels (mg kg ^−1^), with interpolation by towns

	No.^a^	Min	P (25)	P (50)	P (75)	Max
All samples (13317)						
Al		100.000	13,200.000	19,100.000	26,700.000	100,000.000
As		0.100	5.300	9.000	15.300	2510.000
Cd		0.010	0.060	0.100	0.190	17.400
Cr		0.500	15.600	23.200	33.800	2100.000
Cu		0.010	9.140	15.700	25.700	6150.000
Fe		0.010	1.630	2.420	3.380	30.700
Mn		1.000	226.000	384.000	632.000	>10,000.000^b^
Ni		0.100	13.300	22.500	32.100	3840.000
Pb		0.010	15.300	21.400	31.300	9120.000
Zn		0.100	33.100	51.600	75.900	>10,000.000^b^
Interpolation by towns	7917					
Al		3629.000	16,150.000	19,150.000	23,450.000	62,360.000
As		1.000	9.106	12.810	16.970	99.374
Cd		0.016	0.099	0.151	0.231	1.908
Cr		6.458	20.230	24.987	29.860	243.687
Cu		2.676	13.230	17.774	23.450	189.414
Fe		0.325	1.860	2.351	2.881	6.637
Mn		55.033	406.100	510.716	628.500	2272.732
Ni		4.809	19.090	25.213	30.180	502.426
Pb		6.499	18.270	22.612	28.280	682.033
Zn		12.167	44.000	56.050	73.460	457.039

^a^Number of towns

^b^Quantification limit

**Fig. 1:**
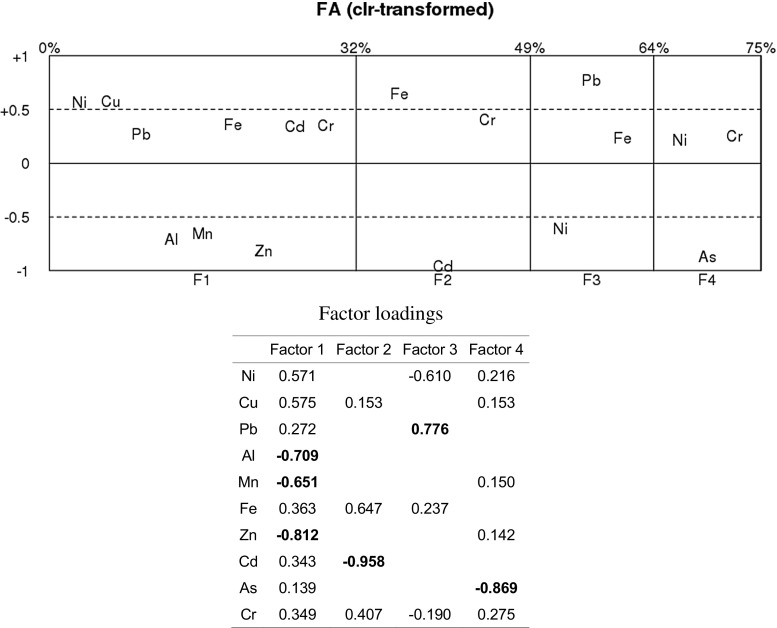
Factor loading plots for the centred logratio-transformed (clr-transformed) four-factor models (PFA and varimax rotation)

Figure 1 shows the factor loading plots for the clr-transformed four-factor models (PFA and varimax rotation). The position of the element names in the plot reflects the loading of that element on the different factors. In addition, the percentages at the top of the plots display the cumulative explained percentage of total variability. The scale on the horizontal axis is in accordance with the relative amount of variability explained by each individual factor (Filzmoser et al. 2009). This figure gives an idea of the significance/composition of each factor. The comments refer to items with factor scores ≥|0.4|. Factor F1 was defined by a combination of negative loadings of Zn, Al, Mn and positive loadings of Ni and Cu. Factor F2 was dominated by negative loadings of Cd and positive loadings of Fe and Cr. Factor F3 increased with the Pb/Ni ratio. Factor F4 decreased when the concentration of As increased, whereas the rest of the composition remained almost constant.

Table 2 shows the relative risks (RRs) with 95% credibility intervals, which do not include unity (statistically significant) of the four score loads for the tumours analysed, by sex. The table shows the associations shared by men and women, with the factors marked in bold. The most evident associations were those displayed by tumours of the digestive system. While the RRs shown were somewhat difficult to interpret due to the transformations, they nonetheless indicate that an increase in factor scores amounts to an increase in mortality. Associations were also observed in the case of haematological tumours.

**Table 2:** Estimates of the effect (RR > 1 and 95% credibility interval (CI)) of factors corresponding to score loads from PFA, on mortality due to different tumour types, by sex. The table shows the results of the clr-transformed data analysis adjusted for sociodemographic variables

Cancer site	Factors	Men			Women		
		RR	95%	CI	RR	95%	CI
Buccal cavity and pharynx	**F1**	1.031	1.009	1.054	1.059	1.023	1.095
Lung	**F1**	1.085	1.018	1.155	1.153	1.072	1.240
NHL	F1	1.010	0.987	1.032	1.022	1.000	1.044
Leukaemias	F1	1.023	1.006	1.041	0.998	0.979	1.017
Oesophagus	F2	0.984	0.960	1.009	1.063	1.008	1.120
Stomach	**F2**	1.051	1.032	1.069	1.043	1.022	1.064
Colorectal	**F2**	1.015	1.003	1.027	1.018	1.005	1.030
Lung	**F2**	1.026	1.015	1.037	1.033	1.012	1.055
Skin	F2	1.005	0.956	1.056	1.084	1.029	1.142
NHL	**F2**	1.027	1.001	1.054	1.038	1.012	1.065
Buccal cavity and pharynx	F3	1.056	1.039	1.072	1.021	0.996	1.046
Oesophagus	**F3**	1.063	1.046	1.081	1.064	1.030	1.099
Stomach	F3	1.013	1.000	1.025	1.001	0.988	1.015
Colorectal	F3	1.008	1.000	1.016	0.986	0.978	0.994
Liver	**F3**	1.053	1.033	1.072	1.057	1.028	1.087
Nasal cavity	F3	1.108	1.043	1.178	1.009	0.931	1.094
Pancreas	**F3**	1.020	1.008	1.032	1.015	1.003	1.028
Lung	**F3**	1.016	1.008	1.024	1.023	1.010	1.037
Pleura	F3	1.059	1.010	1.109	1.028	0.976	1.082
Connective tissue	F3	1.036	1.007	1.067	1.019	0.983	1.057
NHL	**F3**	1.027	1.009	1.044	1.021	1.005	1.038
Leukaemia	F3	1.020	1.007	1.033	1.003	0.990	1.017
Liver	F4	1.001	0.981	1.022	1.065	1.033	1.099
Gallbladder	**F4**	1.031	1.002	1.061	1.049	1.027	1.072
Pleura	F4	1.030	0.973	1.090	1.101	1.024	1.182
Breast	F4				1.014	1.005	1.024

F1: (–Zn –Al –Mn) Ni Cu Fe Cd Cr

F2: (–Cd) Fe Cr

F3: Pb (–Ni)

F4: (–As)

Digestive system tumours displayed the greatest number of associations with factors. The single factor most frequently positively associated with cancer mortality was factor F3 (Pb). It is also important to consider the results of negative associations shown in Table 3. Thus, in the case of factor F4, characterised by a very low As concentration, related mortality was lower than expected for the following tumours: buccal cavity and pharynx; oesophagus; stomach; colorectal; pancreas; prostate; kidney; brain and NHL (see supplementary data).

**Table 3:** Summary of estimates of the protective effect (RR < 1) of factors corresponding to score loads from PFA, on mortality due to different tumour types, by sex. The table shows the results of the clr-transformed data adjusted for sociodemographic variables

Cancer site	Factors	Men			Women		
		RR	95%	CI	RR	95%	CI
Stomach	**F1**	0.966	0.951	0.982	0.948	0.932	0.965
Skin	F1	0.959	0.917	1.002	0.940	0.898	0.984
Melanoma	F2	0.933	0.899	0.969	0.972	0.933	1.013
Uterus	**F2**				0.977	0.957	0.997
Bladder	F2	0.976	0.960	0.993	0.979	0.952	1.006
Myeloma	F2	0.986	0.960	1.013	0.948	0.923	0.974
Bone	F3	1.001	0.967	1.036	0.948	0.911	0.985
Breast	**F3**				0.987	0.978	0.995
Ovarian	**F3**				0.980	0.968	0.992
Buccal cavity and pharynx	F4	0.951	0.934	0.967	0.993	0.963	1.023
Oesophagus	F4	0.970	0.952	0.988	1.011	0.972	1.052
Stomach	**F4**	0.955	0.943	0.968	0.951	0.937	0.966
Colorectal	**F4**	0.977	0.968	0.986	0.990	0.981	0.999
Pancreas	F4	0.972	0.959	0.985	0.987	0.973	1.001
Nasal cavity	F4	0.875	0.812	0.943	0.934	0.838	1.041
Lung	**F4**	0.990	0.982	0.998	0.983	0.968	0.998
Skin	F4	0.980	0.944	1.018	0.939	0.903	0.977
Prostate	**F4**	0.979	0.969	0.988			
Kidney	F4	0.977	0.959	0.995	1.011	0.987	1.035
Brain	F4	0.969	0.952	0.985	0.980	0.961	1.000
NHL	F4	0.975	0.957	0.994	0.983	0.964	1.001

F1: (–Zn –Al –Mn) Ni Cu Fe Cd Cr

F2: (–Cd) Fe Cr

F3: Pb (–Ni)

F4: (–As)

**Fig. 2:**
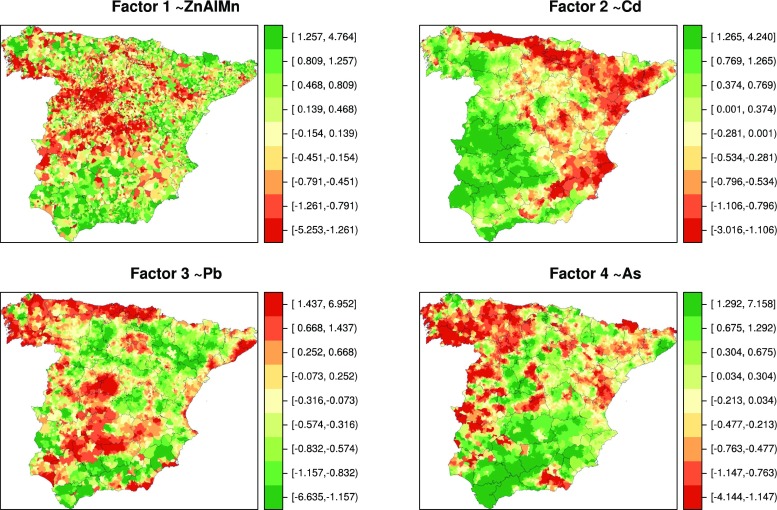
Municipal distribution of score loads from principal factor analysis of heavy metal concentrations in topsoil in mainland Spain. Factorial analysis performed with clr-transformed data

Figure 2 shows maps that plot the municipal distribution of factor scores, with F1 displaying a diffuse spatial pattern marked by a greater presence in inland towns, F2 mostly representing Cd levels, F3 similarly depicting Pb levels, and F4 being characterised by negative factor scores for As.

## Discussion

The results indicate a statistical association between low heavy metal and metalloid content in topsoil and mortality due to tumours of the digestive system in mainland Spain. This association was shown in both sexes, a finding that would support the hypothesis that the incorporation of heavy metals into the trophic chain might be playing a role in the aetiology of some types of cancer. Note should also be taken of the results pertaining to lung and pleural cancer. Similarly, an association with haematological tumours was also in evidence.

With regard to the statistical associations found, F3, characterised by a higher lead versus nickel content, was the single factor that showed the highest number of statistical associations with different tumour locations. Hence, mortality was correlated with this factor in virtually all sites of cancer of the digestive system in men, and in cancer of the oesophagus, liver, pancreas, lung and NHL in both sexes. Some of these associations have been observed by other types of studies in the case of cancer of the stomach (Zhao et al. 2014) and pancreas (Amaral et al. 2012). The IARC classifies inorganic lead compounds as probably carcinogenic to humans (Group 2A) and lead exposure is known to increase the risk of lung, stomach and bladder cancer (IARC Working Group 2006).

A result of considerable interest was that pertaining to As. Factor F4 was fundamentally characterised by low content in arsenic with respect to other metals (As factor loadings of −0.87). Cancers that displayed RRs which were less than 1 and were statistically significant for factor F4, in both sexes, were those of buccal cavity and pharynx, oesophagus, stomach, colorectal, pancreas, lung, prostate, kidney, brain and NHL. The converse of these results would support the possible role of topsoil As in cancer, as previously reported elsewhere (Nuñez et al. 2016).

With regard to the RRs magnitude, the clr-transformations of data and the use of factorial loadings, make it difficult to measure the impact on the population. The risk of the population derived from the composition of the soil is probably very low, but it is surprising to find the displayed spatial dependence.

The concentration of heavy metals in soil determines their presence in animal tissue (López Alonso et al. 2002). The small number of studies means that there is very little epidemiological evidence of the association between arsenic topsoil levels and frequency of cancer. However, heavy metal and arsenic topsoil concentrations serve as an indicator of long-term exposure to these elements (Tchounwou et al. 2012). Exposure to arsenic and inorganic arsenic compounds cause, with sufficient evidence, lung, skin and urinary bladder cancers and with limited evidence kidney, liver and prostate cancer. Cadmium and cadmium compounds exposure is a cause of lung cancer and possible of prostate and kidney cancers. Chromium (VI) compounds are a cause of lung cancer and possibly nasal cavity and paranasal sinuses cancers. Exposure to nickel compounds is a cause of lung, nasal cavity and paranasal sinuses cancers. The biological mechanisms involved are oxidative DNA damage, genomic instability, aneuploidy, gene amplification, epigenetic effects, DNA-repair inhibition leading to mutagenesis among others (Straif et al. 2009).

It is of interest to view the results from the perspective of each type of cancer and find that the tumours which displayed most associations with the respective factors were stomach and lung cancer (see supplementary material): while stomach cancer was associated with all four factors, i.e. F1, F2 and F4 in both sexes and F3 in men only, lung cancer was associated with F2, F3 and F4 in both sexes.

The risk resulting from chronic exposure to a mixture of potentially toxic items in topsoil confronts one with the problem of choosing a paradigm different to the prevailing one of “single chemical as carcinogen” (Miller et al. 2016). Regulatory measures governing tolerable/permitted-to-toxic environmental levels are based on this paradigm, and its reconsideration has thus to be based on a process of proper evaluation/testing which highlights the importance of mixtures of toxic elements.

The mechanism underlying the results obtained for tumours of the digestive system and haematological tumours might be the incorporation of these elements into trophic chain and drinking water, since diet is the main source of exposure to potentially toxic elements (Delgado-Andrade et al. 2003) (Burló et al. 2012).

Metals of the soil are hardly degradable by microbes of the soil and its extension to the food chain poses a threatens potential for the health human (Li et al. 2014). Human beings could be exposed to heavy metals from soils via several pathways, ingestion (particles, diet and water), dermal contact and inhalation (Abrahams 2002) (Liu et al. 2013).

One limitation of this type of studies lies in their lack of speciation of the element. Speciation determines the potential bioaccessibility and bioavailability of the element. Arsenic, for example, is in the environment such as organic and inorganic compounds and the problem of toxicity is with inorganic compounds (Kamel Boulos and Le Blond 2016). The two forms of inorganic arsenic, reduced (trivalent As (III)) and oxidised (pentavalent As (V)), can be absorbed, and accumulated in tissues and body fluids and are the most common oxidation states. Trivalent are more toxic than pentavalent inorganic arsenic species, and while pentavalent arsenicals may be more likely to occur in natural environments, following absorption into the body, pentavalent arsenicals may be reduced to methylated trivalent metabolites, and the overall toxicity is dependent on the rate of methylation of the As (III) formed (Vahter and Concha 2001).

Our results raise the importance of the compositional nature of data in this type of analysis, e.g. the regression analysis based on clr-transformation allowed us to assess the relationship with different mixtures/cocktails of metals (relative proportion of each element in the factor) (Aitchison 2003) (Reimann et al. 2011) (van den Boogaart and Tolosana-Delgado 2013). The absolute magnitudes of compositional variables are ratios to a common sum (in our case 1 Kg). Thus, no single variable is free to vary separately from the rest of the composition. However, the relative magnitudes are the ratio of each compositional variable compared to a single variable and remove the closure effect. The centred logratio (clr) is such a transformation and provide a one-to-one relationship from the simplex to the standard Euclidean space with good geometric properties. The direct application of multivariate statistical methods to raw compositional data can lead to improper results. For example, the problem of so-called spurious correlations occurs, namely that one can obtain different results of correlation analysis, depending on whether the whole composition or only a subcomposition is taken (Filzmoser et al. 2009). However, these are methods which were developed in geochemistry and which are only just beginning to become known in the epidemiological sphere. While examples of compositional data analysis were indeed found in epidemiology, and specifically in nutrition and microbiota studies, it is nonetheless acknowledged that there has been insufficient methodological development in this area (Leite 2014) (Tsilimigras and Fodor 2016).

Spatial models were used in the analysis, thus making it possible to estimate the overall influence of topsoil trace components on mortality. Furthermore, the estimates of RR of death were adjusted for various sociodemographic variables. Generally speaking, this type of analysis tends to be somewhat conservative (relatively insensitive), and revealing associations proves difficult (Richardson et al. 2004).

However, we have to be very careful in the interpretation of these results since the statistical associations, although they seem plausible, do not mean a direct biological correlate. The results support the interest of inclusion of heavy metal levels in topsoil as a hypothesis in analytical epidemiological studies of cancer using biological markers of exposure to heavy metals and metalloids.

## Conclusions

The results of our study show a statistical association between the trace contents of heavy metals and metalloids in topsoil and mortality due to tumours of the digestive system in mainland Spain. This association is observed in both sexes, a finding which would support the hypothesis that the incorporation of heavy metals into the trophic chain might be playing a role in the aetiology of some types of cancer. Similarly, noteworthy are the results pertaining to lung cancer and pleural cancer and the association with haematological tumours. Accordingly, the presence of potentially toxic elements in trace concentrations in topsoil composition might be an additional component in the aetiology of some types of cancer, and go some way to determine the ensuing geographical differences in Spain. The results support the interest of inclusion of heavy metal levels in topsoil as a hypothesis in analytical epidemiological studies using biological markers of exposure to heavy metals and metalloids.

## Electronic supplementary material

Supplementary Table S1 (DOCX 18 kb)
