# Supplementary material for: Association between heavy metal and metalloid levels in topsoil and cancer mortality in Spain
Source: Environ Sci Pollut Res Int. 2017 Jan 20;24(8):7413–21. doi: 10.1007/s11356-017-8418-6 (PMC5383678; doi:10.1007/s11356-017-8418-6)
Supplement: Supplementary file 1 — (DOCX 18 kb) [file 11356_2017_8418_MOESM1_ESM.docx]

**Supplementary material**

**Association between heavy metal and metalloid levels in topsoil and cancer mortality in Spain**

Table S1. Number of deaths by different studied tumours produced in continental Spain between the years 1999-2008.

| **Cancer site** | **ICD-9** | **ICD-10** | **Deaths Men** | **Deaths Women** | **Total** |
| --- | --- | --- | --- | --- | --- |
| Buccal cav. and pharynx | C00-C14 | 140-149 | 16274 | 3761 | 20035 |
| Esophagus | C15 | 150 | 14287 | 2228 | 16515 |
| Stomach | C16 | 151 | 34679 | 21692 | 56371 |
| Colorectal | C18-C21 | 153-154,159.0 | 68353 | 51310 | 119663 |
| Liver | C22.0 | 155.0 | 16994 | 6271 | 23265 |
| Gallbladder | C23-C24 | 156 | 4528 | 8005 | 12533 |
| Pancreas | C25 | 157 | 22458 | 19816 | 42274 |
| Peritoneum | C45.1.C48 | 158 | 964 | 1189 | 2153 |
| Nasal cavity | C30-C31 | 160 | 613 | 266 | 879 |
| Larynx | C32 | 161 | 15110 | 609 | 15719 |
| Lung | C33-C34 | 162 | 155142 | 21657 | 176799 |
| Pleura | C38.4,C45.0 | 163 | 1599 | 650 | 2249 |
| Bone | C40-C41 | 170 | 1464 | 1124 | 2588 |
| Connective tissue | C49 | 171 | 2102 | 1957 | 4059 |
| Melanoma | C43 | 172 | 4003 | 3356 | 7359 |
| Skin | C44 | 173 | 2295 | 2035 | 4330 |
| Breast | C50 | 174 | - | 54887 | 54887 |
| Uterus | C53-C55 | 179-182 | - | 17117 | 17117 |
| Ovarian | C56,C57 | 183 | - | 17768 | 17768 |
| Prostate | C61 | 185 | 52528 | - | 52528 |
| Bladder | C67 | 188 | 33632 | 7212 | 40844 |
| Kidney | C64-C66,C68 | 189 | 11509 | 5854 | 17363 |
| Brain | C71 | 191 | 12371 | 9881 | 22252 |
| Thyroid | C73 | 193 | 880 | 1696 | 2576 |
| NHL | C82-C85,C96 | 200,202 | 11706 | 10499 | 22205 |
| Myeloma | C90 | 203 | 7308 | 7450 | 14758 |
| Leukemias | C91-C95 | 204-208 | 15861 | 12376 | 28237 |

Table S2. Summary of estimates of the effect (RR) of factors corresponding to score loads from PCA, on mortality due to different tumour types, by sex. The table shows the results of the **clr-transformed** data adjusted for socio-demographic variables.

|  |  |  |  | **Men** |  |  |  | **Women** |  |
| --- | --- | --- | --- | --- | --- | --- | --- | --- | --- |
| **Cancer site** | **Factors** |  | **RR** | **95%** | **CI** |  | **RR** | **95%** | **CI** |
|  |  |  |  |  |  |  |  |  |  |
| **Buccal cavity and pharynx** | **F1** |  | **1.031** | **1.009** | **1.054** |  | **1.059** | **1.023** | **1.095** |
|  | F2 |  | 0.976 | 0.952 | 0.999 |  | 0.985 | 0.946 | 1.025 |
|  | F3 |  | **1.056** | **1.039** | **1.072** |  | 1.021 | 0.996 | 1.046 |
|  | F4 |  | 0.951 | 0.934 | 0.967 |  | 0.993 | 0.963 | 1.023 |
|  |  |  |  |  |  |  |  |  |  |
| **Oesophagus** | F1 |  | 1.019 | 0.996 | 1.043 |  | 1.098 | 1.047 | 1.152 |
|  | F2 |  | 0.984 | 0.960 | 1.009 |  | **1.063** | **1.008** | **1.120** |
|  | **F3** |  | **1.063** | **1.046** | **1.081** |  | **1.064** | **1.030** | **1.099** |
|  | F4 |  | 0.97 | 0.952 | 0.988 |  | 1.011 | 0.972 | 1.052 |
|  |  |  |  |  |  |  |  |  |  |
| **Stomach** | F1 |  | 0.966 | 0.951 | 0.982 |  | 0.948 | 0.932 | 0.965 |
|  | **F2** |  | **1.051** | **1.032** | **1.069** |  | **1.043** | **1.022** | **1.064** |
|  | F3 |  | **1.013** | **1.000** | **1.025** |  | 1.001 | 0.988 | 1.015 |
|  | F4 |  | 0.955 | 0.943 | 0.968 |  | 0.951 | 0.937 | 0.966 |
|  |  |  |  |  |  |  |  |  |  |
| **Colorectal** | F1 |  | 1.009 | 0.998 | 1.019 |  | 0.988 | 0.977 | 0.998 |
|  | **F2** |  | **1.015** | **1.003** | **1.027** |  | **1.018** | **1.005** | **1.030** |
|  | F3 |  | **1.008** | **1.000** | **1.016** |  | 0.986 | 0.978 | 0.994 |
|  | F4 |  | 0.977 | 0.968 | 0.986 |  | 0.990 | 0.981 | 0.999 |
|  |  |  |  |  |  |  |  |  |  |
| **Liver** | F1 |  | 1.021 | 0.996 | 1.047 |  | 1.029 | 0.992 | 1.068 |
|  | F2 |  | 0.991 | 0.964 | 1.019 |  | 1.024 | 0.981 | 1.069 |
|  | **F3** |  | **1.053** | **1.033** | **1.072** |  | **1.057** | **1.028** | **1.087** |
|  | F4 |  | 1.001 | 0.981 | 1.022 |  | **1.065** | **1.033** | **1.099** |
|  |  |  |  |  |  |  |  |  |  |
| **Gallbladder** | F1 |  | 0.996 | 0.964 | 1.029 |  | 1.004 | 0.978 | 1.031 |
|  | F2 |  | 0.976 | 0.94 | 1.014 |  | 0.95 | 0.923 | 0.978 |
|  | F3 |  | 0.981 | 0.958 | 1.006 |  | 0.957 | 0.939 | 0.975 |
|  | **F4** |  | **1.031** | **1.002** | **1.061** |  | **1.049** | **1.027** | **1.072** |
|  |  |  |  |  |  |  |  |  |  |
| **Pancreas** | F1 |  | 0.981 | 0.966 | 0.996 |  | 0.99 | 0.974 | 1.006 |
|  | F2 |  | 0.999 | 0.982 | 1.017 |  | 0.992 | 0.974 | 1.011 |
|  | **F3** |  | **1.020** | **1.008** | **1.032** |  | **1.015** | **1.003** | **1.028** |
|  | F4 |  | 0.972 | 0.959 | 0.985 |  | 0.987 | 0.973 | 1.001 |
|  |  |  |  |  |  |  |  |  |  |
| **Peritoneum** | F1 |  | 1.050 | 0.981 | 1.123 |  | 0.962 | 0.912 | 1.015 |
|  | F2 |  | 0.941 | 0.867 | 1.020 |  | 1.026 | 0.958 | 1.098 |
|  | F3 |  | 1.027 | 0.977 | 1.080 |  | 1.006 | 0.968 | 1.045 |
|  | F4 |  | 0.962 | 0.903 | 1.024 |  | 0.999 | 0.950 | 1.051 |
|  |  |  |  |  |  |  |  |  |  |
| **Nasal cavity** | F1 |  | 0.969 | 0.895 | 1.049 |  | 0.912 | 0.814 | 1.021 |
|  | F2 |  | 1.067 | 0.968 | 1.176 |  | 1.065 | 0.921 | 1.231 |
|  | F3 |  | **1.108** | **1.043** | **1.178** |  | 1.009 | 0.931 | 1.094 |
|  | F4 |  | 0.875 | 0.812 | 0.943 |  | 0.934 | 0.838 | 1.041 |
|  |  |  |  |  |  |  |  |  |  |
| **Larynx** | F1 |  | 0.998 | 0.976 | 1.020 |  | 1.023 | 0.949 | 1.103 |
|  | F2 |  | 1.002 | 0.978 | 1.026 |  | 1.032 | 0.938 | 1.134 |
|  | F3 |  | 0.997 | 0.982 | 1.014 |  | 1.005 | 0.953 | 1.060 |
|  | F4 |  | 0.993 | 0.975 | 1.010 |  | 1.061 | 0.988 | 1.140 |
|  |  |  |  |  |  |  |  |  |  |
| **Lung** | F1 |  | 0.999 | 0.988 | 1.009 |  | 1.014 | 0.996 | 1.032 |
|  | **F2** |  | **1.026** | **1.015** | **1.037** |  | **1.033** | **1.012** | **1.055** |
|  | **F3** |  | **1.016** | **1.008** | **1.024** |  | **1.023** | **1.010** | **1.037** |
|  | F4 |  | 0.990 | 0.982 | 0.998 |  | 0.983 | 0.968 | 0.998 |
|  |  |  |  |  |  |  |  |  |  |
| **Pleura** | **F1** |  | **1.085** | **1.018** | **1.155** |  | **1.153** | **1.072** | **1.240** |
|  | F2 |  | 0.859 | 0.796 | 0.927 |  | 0.971 | 0.884 | 1.066 |
|  | F3 |  | **1.059** | **1.01** | **1.109** |  | 1.028 | 0.976 | 1.082 |
|  | F4 |  | 1.030 | 0.973 | 1.090 |  | **1.101** | **1.024** | **1.182** |
|  |  |  |  |  |  |  |  |  |  |
| **Bone** | F1 |  | 1.005 | 0.958 | 1.055 |  | 1.026 | 0.971 | 1.085 |
|  | F2 |  | 0.975 | 0.918 | 1.035 |  | 0.995 | 0.929 | 1.066 |
|  | F3 |  | 1.001 | 0.967 | 1.036 |  | 0.948 | 0.911 | 0.985 |
|  | F4 |  | 0.981 | 0.937 | 1.026 |  | 1.017 | 0.965 | 1.071 |
|  |  |  |  |  |  |  |  |  |  |
| **Connective tissue** | F1 |  | 1.016 | 0.976 | 1.057 |  | 0.993 | 0.946 | 1.044 |
|  | F2 |  | 1.032 | 0.981 | 1.086 |  | 0.985 | 0.929 | 1.043 |
|  | F3 |  | **1.036** | **1.007** | **1.067** |  | 1.019 | 0.983 | 1.057 |
|  | F4 |  | 0.998 | 0.961 | 1.036 |  | 1.003 | 0.96 | 1.047 |
|  |  |  |  |  |  |  |  |  |  |
| **Melanoma** | F1 |  | 1.009 | 0.979 | 1.039 |  | 1.015 | 0.983 | 1.048 |
|  | F2 |  | 0.933 | 0.899 | 0.969 |  | 0.972 | 0.933 | 1.013 |
|  | F3 |  | 1.014 | 0.993 | 1.036 |  | 1.013 | 0.990 | 1.037 |
|  | F4 |  | 0.995 | 0.967 | 1.024 |  | 1.002 | 0.971 | 1.033 |
|  |  |  |  |  |  |  |  |  |  |
| **Skin** | F1 |  | 0.959 | 0.917 | 1.002 |  | 0.940 | 0.898 | 0.984 |
|  | F2 |  | 1.005 | 0.956 | 1.056 |  | **1.084** | **1.029** | **1.142** |
|  | F3 |  | 0.984 | 0.951 | 1.017 |  | 0.980 | 0.946 | 1.015 |
|  | F4 |  | 0.980 | 0.944 | 1.018 |  | 0.939 | 0.903 | 0.977 |
|  |  |  |  |  |  |  |  |  |  |
| **Breast** | F1 |  |  |  |  |  | 1.006 | 0.995 | 1.017 |
|  | F2 |  |  |  |  |  | 0.988 | 0.976 | 1.001 |
|  | F3 |  |  |  |  |  | 0.987 | 0.978 | 0.995 |
|  | F4 |  |  |  |  |  | **1.014** | **1.005** | **1.024** |
|  |  |  |  |  |  |  |  |  |  |
| **Uterus** | F1 |  |  |  |  |  | 1.016 | 0.998 | 1.034 |
|  | F2 |  |  |  |  |  | 0.977 | 0.957 | 0.997 |
|  | F3 |  |  |  |  |  | 0.991 | 0.978 | 1.004 |
|  | F4 |  |  |  |  |  | 0.996 | 0.981 | 1.011 |
|  |  |  |  |  |  |  |  |  |  |
| **Ovarian** | F1 |  |  |  |  |  | 0.995 | 0.978 | 1.012 |
|  | F2 |  |  |  |  |  | 0.989 | 0.970 | 1.009 |
|  | F3 |  |  |  |  |  | 0.980 | 0.968 | 0.992 |
|  | F4 |  |  |  |  |  | 0.999 | 0.984 | 1.013 |
|  |  |  |  |  |  |  |  |  |  |
| **Prostate** | F1 |  | 0.992 | 0.980 | 1.003 |  |  |  |  |
|  | F2 |  | 1.000 | 0.987 | 1.013 |  |  |  |  |
|  | F3 |  | 1.001 | 0.992 | 1.010 |  |  |  |  |
|  | F4 |  | 0.979 | 0.969 | 0.988 |  |  |  |  |
|  |  |  |  |  |  |  |  |  |  |
| **Bladder** | F1 |  | 1.006 | 0.991 | 1.021 |  | 1.003 | 0.980 | 1.026 |
|  | F2 |  | 0.976 | 0.960 | 0.993 |  | 0.979 | 0.952 | 1.006 |
|  | F3 |  | 0.993 | 0.982 | 1.004 |  | 0.986 | 0.970 | 1.003 |
|  | F4 |  | 1.01 | 0.997 | 1.022 |  | 0.996 | 0.975 | 1.017 |
|  |  |  |  |  |  |  |  |  |  |
| **Kidney** | F1 |  | 1.012 | 0.991 | 1.033 |  | 0.986 | 0.960 | 1.013 |
|  | F2 |  | 0.995 | 0.971 | 1.019 |  | 0.997 | 0.966 | 1.029 |
|  | F3 |  | 1.014 | 0.998 | 1.030 |  | 1.006 | 0.987 | 1.027 |
|  | F4 |  | 0.977 | 0.959 | 0.995 |  | 1.011 | 0.987 | 1.035 |
|  |  |  |  |  |  |  |  |  |  |
| **Brain** | F1 |  | 1.004 | 0.985 | 1.023 |  | 1.020 | 0.998 | 1.042 |
|  | F2 |  | 0.990 | 0.968 | 1.012 |  | 0.963 | 0.938 | 0.988 |
|  | F3 |  | 1.01 | 0.996 | 1.024 |  | 0.998 | 0.982 | 1.014 |
|  | F4 |  | 0.969 | 0.952 | 0.985 |  | 0.980 | 0.961 | 1.000 |
|  |  |  |  |  |  |  |  |  |  |
| **Thyroid** | F1 |  | 0.943 | 0.887 | 1.003 |  | 1.002 | 0.953 | 1.052 |
|  | F2 |  | 0.967 | 0.895 | 1.045 |  | 0.987 | 0.931 | 1.047 |
|  | F3 |  | 1.003 | 0.960 | 1.048 |  | 1.042 | 1.004 | 1.083 |
|  | F4 |  | 0.952 | 0.898 | 1.010 |  | 0.972 | 0.928 | 1.019 |
|  |  |  |  |  |  |  |  |  |  |
| **NHL** | F1 |  | 1.010 | 0.987 | 1.032 |  | **1.022** | **1.000** | **1.044** |
|  | **F2** |  | **1.027** | **1.001** | **1.054** |  | **1.038** | **1.012** | **1.065** |
|  | **F3** |  | **1.027** | **1.009** | **1.044** |  | **1.021** | **1.005** | **1.038** |
|  | F4 |  | 0.975 | 0.957 | 0.994 |  | 0.983 | 0.964 | 1.001 |
|  |  |  |  |  |  |  |  |  |  |
| **Myeloma** | F1 |  | 1.017 | 0.994 | 1.040 |  | 1.010 | 0.987 | 1.033 |
|  | F2 |  | 0.986 | 0.960 | 1.013 |  | 0.948 | 0.923 | 0.974 |
|  | F3 |  | 1.005 | 0.989 | 1.022 |  | 0.994 | 0.979 | 1.011 |
|  | F4 |  | 0.997 | 0.977 | 1.018 |  | 1.010 | 0.989 | 1.031 |
|  |  |  |  |  |  |  |  |  |  |
| **Leukemias** | F1 |  | **1.023** | **1.006** | **1.041** |  | 0.998 | 0.979 | 1.017 |
|  | F2 |  | 1.019 | 0.998 | 1.039 |  | 1.005 | 0.983 | 1.027 |
|  | F3 |  | **1.020** | **1.007** | **1.033** |  | 1.003 | 0.990 | 1.017 |
|  | F4 |  | 0.999 | 0.984 | 1.014 |  | 1.000 | 0.984 | 1.016 |
